# Reduced fibroblast adhesion and proliferation on plasma-modified titanium surfaces

**DOI:** 10.1007/s10856-014-5278-1

**Published:** 2014-07-24

**Authors:** Sebastian Kuhn, Jennifer Kroth, Ulrike Ritz, Alexander Hofmann, Christian Brendel, Lars Peter Müller, Renate Förch, Pol Maria Rommens

**Affiliations:** 1 Department of Orthopedics and Traumatology, BiomaTiCS Research Group, University Medical Centre of the Johannes Gutenberg University, Langenbeckstr. 1, 55101 Mainz, Germany; 2 Max Planck Institute for Polymer Research, Ackermannweg 10, 55128 Mainz, Germany; 3 Georg Speyer Haus, Paul-Ehrlich-Str. 42-44, 60596 Frankfurt, Germany; 4 Boston Children´s Hospital, 300 Longwood Avenue, Boston, MA 02115 USA; 5 Department for Trauma Surgery, Hand and Elbow Surgery, Centre for Orthopaedic and Trauma Surgery, University Medical Center, Josef-Stelzmann-Str. 9, 50924 Cologne, Germany

## Abstract

Soft tissue complications are clinically relevant problems after osteosynthesis of fractures. The goal is to develop a method for reduction of fibroblast adhesion and proliferation on titanium implant surfaces by plasma polymerisation of the organo-silicon monomer hexamethyldisiloxane (HMDSO). HMDSO was deposited under continuous wave conditions in excess oxygen (ppHMDSO surface) and selected samples were further modified with an additional oxygen plasma (ppHMDSO + O_2_ surface). Surface characterization was performed by scanning electron microscopy, profilometry, water contact angle measurements, infrared reflection absorption spectroscopy and X-ray photoelectron spectroscopy. In our experimental setup the mechanical properties, roughness and topography of the titanium were preserved, while surface chemistry was drastically changed. Fibroblast proliferation was assessed by alamarBlue assay, cell morphology by confocal microscopy visualization of eGFP-transducted fibroblasts, and cell viability by Annexine V/propidium iodide assay. Both modified surfaces, non-activated hydrophobic ppHMDSO and activated hydrophilic ppHMDSO + O_2_ were able to dramatically reduce fibroblast colonization and proliferation compared to standard titanium. However, this effect was more strongly pronounced on the hydrophobic ppHMDSO surface, which caused reduced cell adhesion and prevented proliferation of fibroblasts. The results demonstrate that plasma modifications of titanium using HMDSO are valuable candidates for future developments in anti-adhesive and anti-proliferative coatings for titanium fracture implants.

## Introduction

Titanium implants are used for operative fracture fixation. In the past those implants were mainly designed as mechanical devices and the biological effects were largely ignored. Depending on the site of application different implant characteristics and resulting surface properties are warranted. In situations where tendons are in close proximity to implants, such as in osteosynthesis of hand and wrist fractures, soft-tissue adhesion is a disadvantage [1, 2]. Direct tissue contact and adhesion to the implant may diminish tendon excursion and cause tenosynovitis or even tendon rupture [3, 4]. It has been proposed that a non-adhering fibrous capsule on the soft-tissue side of an osteosynthesis plate may reduce the chance of soft tissues (tendons, muscles and nerves) adhesion to the implant [5]. Fibroblasts are the main cellular constituent of the adjacent soft tissue and therefore warrant the main focus in these investigations. Studying the proliferation, morphology and adhesion of fibroblasts on differently modified titanium surfaces can give an indication of the cyto-compatibility of the surface and its suitability for possible further applications as fracture and orthopaedic implants. While many publications focus on improving cellular attachment and proliferation in order to achieve a more robust soft tissue and bone healing response, far less studies have been published on modifications leading to the reduction of these.

The molecular events at the implant to soft tissue interface are influenced by the surface properties. They include surface chemistry, hydrophilicity/-phobicity, heterogeneity, surface charge, and topography [6]. Among these, topography has so far proven the greatest effect. An “effective roughness spectrum” hypothesis has been proposed [6]. Polished pure titanium and titanium molybdenum alloy implants with reduced surface roughness have demonstrated reduced cell and tissue adhesion in vitro and in vivo [7–9]. Therefore, reducing the implant’s surface micro-roughness is one of various possible logical modifications.

Another possibility, namely the effects of changing the surface chemistry of already existing titanium implants to reduce tissue adhesion, is far less investigated. Plasma modification is an effective and economical surface treatment technique of growing interest in biomedical engineering [10]. The unique advantage of plasma modification is that surface properties and biocompatibility can be engineered selectively, while the bulk properties of the material remain unchanged [11]. The organo-silicon monomer hexamethyldisiloxane (HMDSO), is one of the most studied and exploited precursors in plasma-assisted deposition today due to its easy fabrication, low cost and biocompatibility [10, 12]. By controlling the process parameters it is possible to tailor the physical and chemical properties of the material surface and as a consequence the biomedical behaviour [13, 14]. Films with varying properties ranging from semi-organic SiO_x_C_y_H_z_ to inorganic SiO_2_-like can be deposited by varying the HMDSO to oxygen ratio in the gas mixture [15]. Plasma deposited HMDSO has been studied for coatings in vascular grafts and prosthesis [16]. The films have also gained interest as possible coatings for titanium dental implants [13, 17, 18].

The aim of our study is to modify a standard, clinically used titanium implant surface by HMDSO plasma polymerization, with the goal of reducing fibroblast adhesion and proliferation. In this in vitro study, we produced two HMDSO plasma modified variants (hydrophobic and hydrophilic) of grade 4 titanium. Commercial fibroblasts (NHDF-p adult) were chosen to assess the implant-fibroblast interaction by examining cell proliferation, morphology and viability.

## Materials and methods

### Titanium substrates

Titanium discs were manufactured from commercially pure grade 4 titanium (ISO 5832-2, Medartis, Basel, Switzerland). Discs measuring 14.5 mm in diameter/1.6 mm in thickness and 33.5 mm in diameter/1.6 mm in thickness were used in this study. The diameters were chosen to ensure that the discs would match the diameter of 6- and 24-well plates. The 33.5 mm diameter titanium and HMDSO-modified discs were used for the Annexine V/propidium iodide assay. For all other cell experiments the smaller discs with a diameter of 14.5 mm were used. All production steps including cutting, deburring, and ceramic tumbling were done according to the standard manufacturers protocol for clinical fracture implants to provide an identical surface. These unmodified titanium discs samples served as controls in all experiments. Titanium samples were cleaned first by sonication for 15 min with a 2 % Hellmanex II alkaline cleaning solution (Helma, Müllheim, Germany). After thorough washing with ultrapure water, titanium discs were finally sonicated twice for 15 min with absolute ethanol (Sigma Aldrich Chemie GmbH, Munich, Germany). The samples were dried under a nitrogen stream and directly subjected to plasma polymerization.

### Plasma polymerization

Plasma polymerization of HMDSO films on the titanium discs was carried out using an in-house fabricated capacitively-coupled radio-frequency plasma reactor at the Max Planck Institute for Polymer Research (Mainz, Germany). It consists of a 30 cm long and 10 cm in diameter cylindrical Pyrex glass tube, equipped with two concentric metal rings located outside the reactor and separated about 12 cm, which deliver 13.56 MHz radio frequency to the chamber. Prior to deposition of the HMDSO films, the reactor chamber was cleaned using an argon (30 sccm)/oxygen (10 sccm) gas plasma run at an input power of 150 W for at least 15 min. The substrates were always placed in the centre of the plasma reactor. The base pressure in the reactor was 1 × 10^−4^ mbar achieved by a rotary vane pump (Oerlikon Leybold Vacuum GmbH, Cologne, Germany). Prior to plasma deposition, titanium substrates were cleaned using an argon (30 sccm)/oxygen (10 sccm) gas plasma run at an input power of 100 W for 3 min. The sample chamber was left under vacuum for another 15 min to allow the evaporation of organic components. HMDSO was always deposited in excess oxygen (10 sccm) and the monomer was degassed by repeated freeze thaw cycles to remove dissolved gasses, prior to use for plasma polymerization. The flow rate of HMDSO was controlled by a needle valve and the total gas pressure was always set to 1 × 10^−1^ mbar for the deposition. In the present work HMDSO was deposited under continuous wave conditions using an input power of 70 W with a deposition time of 10 ± 2 s (ppHMDSO surface). Selected samples (ppHMDSO + O_2_ surface) were further modified with an additional two second long oxygen plasma treatment at 50 W input power and an oxygen flow of 10 sccm. This surface was used for comparison to induce variations in hydrophilicity for the same plasma polymer system.

Additionally “pattern surfaces” were produced under identical conditions, covering part of the disc with an aluminium mask showing several 10 × 2 mm slits. This led to samples showing two different surface properties: either displaying titanium — ppHMDSO or titanium — ppHMDSO + O_2_ on the same substrate surface.

### Scanning electron microscopy (SEM)

The surface morphology was analysed using a Hitachi SU8000 Scanning Electron Microscope (Hitachi High Technologies Europe GmbH, Krefeld, Germany). Images were taken with an accelerating voltage of 0.7 kV for superficial surface imaging and 10 kV for visualization of the underlying surface.

### Surface profilometry

Surface roughness was determined using a surface profiler (P-10 Surface Profiler, KLA-Tencor, Milpitas, USA). Measurements were conducted at a scanning speed of 10 μm/s, a force of 1 mg and a scan frequency of 100 Hz covering a distance of 1,000 μm. For each surface 10 random measurements were performed before calculating the surface roughness.

### Step profilometry

Plasma polymer thickness was measured using a step profilometer (KLA Tencor P 10, Milpitas, USA). Silicon wafers were used as substrates due to the high surface roughness of the titanium disks. During the plasma deposition processes silicon wafer substrates were partly covered with a microscope glass slide. To analyse the thickness, the glass slide was removed and the step height was measured. Measurements were conducted at a scanning speed of 50 μm/s, a force of 1 mg and a scan frequency of 100 Hz covering a distance of 500 μm. Five random measurements were performed for each surface before calculating the film thickness.

### Static contact angle

The surface wetting behaviour was assessed by water contact angle measurements using the sessile drop technique. A drop volume of 4 µl ultrapure water (18.2 MΩ cm^−1^ resistivity, Millipore, Molsheim, France) was delivered at 10 µl/s to the respective surface. On three different samples, five measurements were performed for each surface using a Goniometer (DSA100-MK2, Kruess GmbH, Hamburg, Germany).

### Infrared reflection absorption spectroscopy (IRRAS)

The deposited films were studied by IRRAS using a Thermo Scientific Nicolet Magna-IR 850 (Thermo Scientific Nicolet, West Palm Beach, USA). The samples were irradiated with polarized light at an incidence angle of 5° in the reflectance sampling measurement mode. Measurements were performed on glass slides covered with an 80 nm gold film by thermal evaporation using an Edwards FL-400 electron beam evaporator (Edwards Vacuum, Crawly, UK). For all the IRRAS measurements, Omnic series software (Omnic version 7.0, Thermo Electron Corporation, Waltham, USA) was utilized for data acquisition.

### X-ray photoelectron spectroscopy (XPS)

XPS measurements were carried out using a Perkin Elmer PHI 5600 ci instrument (Physical Electronics, Eden Prairie, USA) equipped with a non-monochromatic Mg Kα X-ray source working at 300 W. Samples were analysed at a take off angle of 45°. The analyses were performed at IMTEK (Department of Microsystems Engineering, Albert-Ludwigs University, Freiburg, Germany).

### Cultivation of fibroblasts

Commercial human dermal fibroblasts (NHDF adult, Promocell, Heidelberg, Germany) were used for all cell experiments. NHDF fibroblasts were cultured in medium containing 72 % Dulbecco’s modified Eagle medium (ATCC, Manassas, USA), 18 % Medium M199 (Sigma-Aldrich, Steinheim, Germany), 9 % fetal calf serum (PAA Laboratories, Pasching, Austria) and 1 % Penicillin–Streptomycin (Invitrogen, Karlsruhe, Germany). Cells were incubated at 37 °C in humidified air with 5 % CO_2_ atmosphere. Culture medium was changed twice a week. Cells were subcultured by detachment with Accutase (PAA Laboratories, Pasching, Austria) at approximately 90 % confluence. Fibroblasts from the 2nd to 6th passage were used for all experiments.

### Cell viability and proliferation

Cell viability and proliferation on the different surfaces were measured using the semi-quantitative colorimetric alamarBlue assay (Invitrogen, Karlsruhe, Germany). The unmodified and modified 14.5 mm titanium discs were placed in 24-well ultra-low attachment plates (Corning Life Sciences, Amsterdam, Netherlands) to assure cell attachment to the titanium surfaces and to avoid cell attachment to the culture plates. The surfaces were seeded with NHDF fibroblasts in 1 ml culture medium at a concentration of 15,000 cells/ml. After 24 h, the medium was removed and replaced by a culture medium solution containing 10 % alamarBlue. The plates were further incubated for 4 h at 37 °C. 100 μl aliquots were transferred into wells of 96 well plates and the absorption difference at 570 nm and 600 nm was measured in a multiplate spectrophotometer (Tecan, Maennedorf, Switzerland). The ongoing assay was performed once every 24 h over a time period of seven days. As a control, alamarBlue was added to the cell growth medium without cells. The assay was performed in quintuplicates and was repeated three times.

### Lentiviral transduction of fibroblasts with pHR′-SEW eGFP vector

Gene transfer of eGFP into NDHF-p adult fibroblasts was achieved by lentiviral transduction according to a published protocol [19]. The eGFP encoding lentiviral vector pHR′-SEW was used to prepare vector supernatants by transfection of 293T cells as previously described [19]. For gene transfer, 15.000 fibroblasts (NHDF adult, Promocell, Heidelberg, Germany) were seeded into 24-well tissue culture plates (Greiner, Frickenhausen, Germany). Two rounds of transduction on day 1 and 3 were performed at a cumulative multiplicity of infection (MOI) of ~100 to achieve >98 % gene marking. Transduction efficiency was confirmed by fluorescence microscopy (Wilovert AFL30, Hundt GmbH, Wetzlar, Germany) and flow cytometry FACSCalibur (BD Biosciences, San Jose, USA) using the CellQuestPro Software (BD Biosciences, San Jose, USA).

### Confocal laser scanning microscopy (CLSM)

For cell visualization the 14.5 mm disks (Titanium, ppHMDSO and ppHMDSO + O_2_) were seeded with 3,000 eGFP-transducted fibroblasts in 1 ml culture medium in 24-well ultra-low attachment plates (Corning Life Sciences, Amsterdam, Netherlands).

CLSM (Leica TCS SP2, Bensheim, Germany) enabled visualization of cells on the respective surfaces. Water immersion lenses allowed cell visualization without removal of the culture medium, which protects the cells against dehydration. Visualization of the eGFP-transducted fibroblasts was conducted by argon-krypton laser excitation at 488 nm and detection from 500 to 550 nm. Samples were imaged at 20× magnification and saved at a 1024 × 1024 pixel resolution with the native Leica software. Imaging was performed from three independent experiments in triplicate each, for every surface and point of time (1, 3 and 5 days). Ten images were taken per disc, which resulted in 90 images per surface modification and each point of time.

### Quantitative cell surface analysis of CLSM images

Surface area covered by fibroblasts and cell circularity were analysed by image processing with ImageJ, Version 1.44 (NIH, Bethesda, USA). CLSM images were imported into ImageJ, and a threshold was selected to differentiate the cells from the background. The covered area and cell circularity were analysed after selecting a minimal particle size of 200 pixels. Per sample, ten images were analysed and the percentage of the total fibroblasts covered area calculated.

### Imaging of patterned surfaces

Imaging of the patterned surfaces was analogue to the CLSM imaging described above. However 14.5 mm disks were seeded with 15,000 eGFP-transducted fibroblasts in 1 ml culture medium in 24-well ultra-low attachment plates (Corning Life Sciences, Amsterdam, Netherlands). Samples were imaged at 5× magnification and saved at a 1,024 × 1,024 pixel resolution with the native Leica software.

### Imaging of cells from the supernatant

To investigate the viability of fibroblasts in the supernatant of the ppHMDSO surface, after 5 days of incubation, the supernatant was transferred to standard TCP (Greiner, Frickenhausen, Germany). CLSM visualisation was conducted on day 10.

### Annexine V/propidium iodide assay

Cell viability, apoptosis and necrosis were assessed by flow cytometry after dual staining with annexin V-FITC and propidium iodide. The 33.5 mm diameter titanium and HMDSO-modified discs were placed in 6-well ultra-low attachment plates (Corning Life Sciences, Amsterdam, Netherlands) and seeded with 150,000 NHDF fibroblasts in 3 ml culture medium. The rate of apoptosis and necrosis was determined at four time-points (6, 12, 24 h and 5 days). Cell culture medium, PBS rinsing solution and cell suspension, obtained through accutase (PAA, Parsing, Germany), were collected. The cells were pelleted at 1,400 rpm for 5 min. Cells were resuspended in 100 μl of buffer (10 mM Hepes, 140 mM NaCl, 5 mM CaCl_2_, pH 7.4) containing 2 μl of FITC-labelled annexin V (Annexin-FLUOS staining kit, Roche Diagnostics, Germany) and 2 µl propidium iodide (50 µg/ml, Applichem, Darmstadt, Germany). The solution was incubated for 20 min in darkness at room temperature. Prior to FACS analysis, 400 µl PBS was added to the solution.

The rate of apoptosis and necrosis of the cells were determined by flow cytometry (FACSCalibur, BD Bioscience, Heidelberg, Germany). The fluorescence intensity of annexin V-FITC (excitation/emission wavelength 488/518 nm, detection wavelength 530 nm, FL-1) versus propidium iodide excitation/emission wavelength 535/617 nm, detection wavelength: 670 nm, FL-3) was recorded. For each sample, 10,000 events were acquired. Dual analysis of the obtained data was performed using the BD Software CellQuest Pro Version 2.0 to distinguish viable from apoptotic and necrotic cells. The assay was performed in triplicate for each surface at the four time-points.

### Statistical analysis

Statistical analysis was conducted using SPSS 19.0 for Windows (IBM, Armonk, New York, United States). Means and standard deviations (SD) were calculated for descriptive analysis.

For confirmatory data analysis alamarBlue proliferation assay and cell surface area were analysed at each time-point with the two-tailed Friedman test as a non-parametrical statistical test. A global significance level for all statistical tests procedures conducted was chosen to *α* = 0.05. Due to multiple testing the Bonferroni correction (*α/n*, where *n* is the number of statistical tests, which are analysed confirmatory) was performed. Seven hypotheses (day 1–day 7) were analysed for the alamarBlue proliferation assay. For the cell surface analysis three hypotheses (day 1, 3 and 5) were analysed. Therefore every Friedman test was performed to the local significance level of *α/n* = 0.05/7 = 0.007 in the data analysis of the alamarBlue assay and to a local significance level of *α/n* = 0.05/3 = 0.0167 for cell surface analysis.

Paired analysis was performed at each time-point between the three surfaces with the two tailed non-parametric Wilcoxon signed-rank test. These pairwise analyses were regarded as explorative, and the *P* *-*values of the corresponding tests are presented for descriptive reasons.

## Results

### Scanning electron microscopy (SEM)

**Fig. 1:**
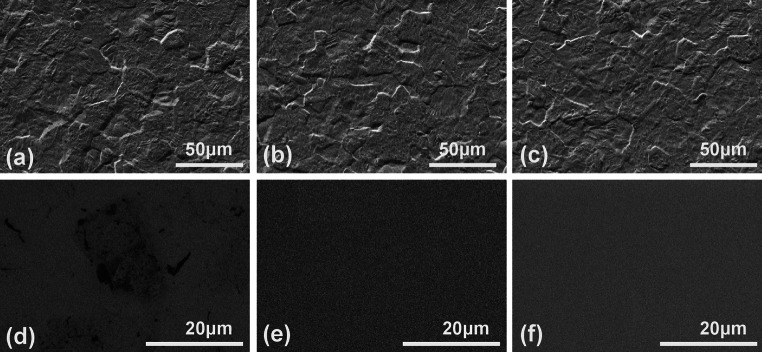
SEM imaging for titanium (**a**,**d**), ppHMDSO (**b**,**e**) and ppHMDSO + O_2_ (**c**,**f**). Images (**a**–**c**) were taken with an accelerating voltage of 10 kV for visualization of the underlying surface and images (**d**–**f**) with 0.7 kV for superficial surface imaging

SEM images for the unmodified titanium substrates demonstrated the typical surface properties of commercially pure grade 4 titanium (Fig. 1a). It displays the typical “basket weave” topography, with numerous peaks and troughs. Titanium surface modification with ppHMDSO and (Fig. 1b) ppHMDSO + O_2_ (Fig. 1c) did not lead to a detectable change of the surface topography on SEM imaging. In comparison to the unmodified titanium (Fig. 1d), superficial 0.7 kV SEM imaging demonstrated homogenous surface coating for ppHMDSO (Fig. 1e) and ppHMDSO + O_2_ (Fig. 1f).

### Surface profilometry

Plasma polymerization with HMDSO did not change the surface roughness of the grade 4 titanium materials. The variation in surface roughness values was within the typical range for this method. Detailed results are shown in Table 1.

**Table 1:** Results of the surface characterization of titanium, ppHMDSO and ppHMDSO + O_2_

	Titanium	ppHMDSO	ppHMDSO + O_2_
Plasma modification (s)			
HMDSO Plasma	–	10 ± 2	10 ± 2
Additional O_2_ plasma	–	–	2
Surface roughness (Ra) mean ± SD (nm)	251 ± 17	266 ± 25	242 ± 23
Film thickness mean ± SD (nm)	–	54.1 ± 14.2	50.2 ± 10.0
Contact angle mean ± SD (°)	49° ± 3	96° ± 2	37° ± 3
Elemental surface composition (XPS) mean ± SD (at. %)			
Titanium	16.9 ± 0.8		
Carbon	27.0 ± 2.4	42.1 ± 0.5	21.9 ± 1.0
Oxygen	52.3 ± 1.7	31.8 ± 0.1	49.7 ± 0.9
Silicone	3.8 ± 1.2	26.2 ± 0.4	28.3 ± 0.2
O/C	–	0.8	2.7
O/Si	–	1.2	1.8
Si_x_O_y_C_z_	–	Si_1_O_1.2_C_1.6_	Si_1_O_1.8_C_0.8_

### Step profilometry

The plasma polymer film thickness was measured using a step profilometer and demonstrated for both HMDSO modifications a thickness of approximately 50 nm. Detailed results are shown in Table 1.

### Static contact angle

The standard titanium and titanium discs modified with ppHMDSO films showed typical static contact angles. The order of the surfaces from hydrophilic to hydrophobic was ppHMDSO + O_2_, titanium and ppHMDSO. Detailed results are shown in Table 1.

### Infrared reflection absorption spectroscopy (IRRAS)

**Fig. 2:**
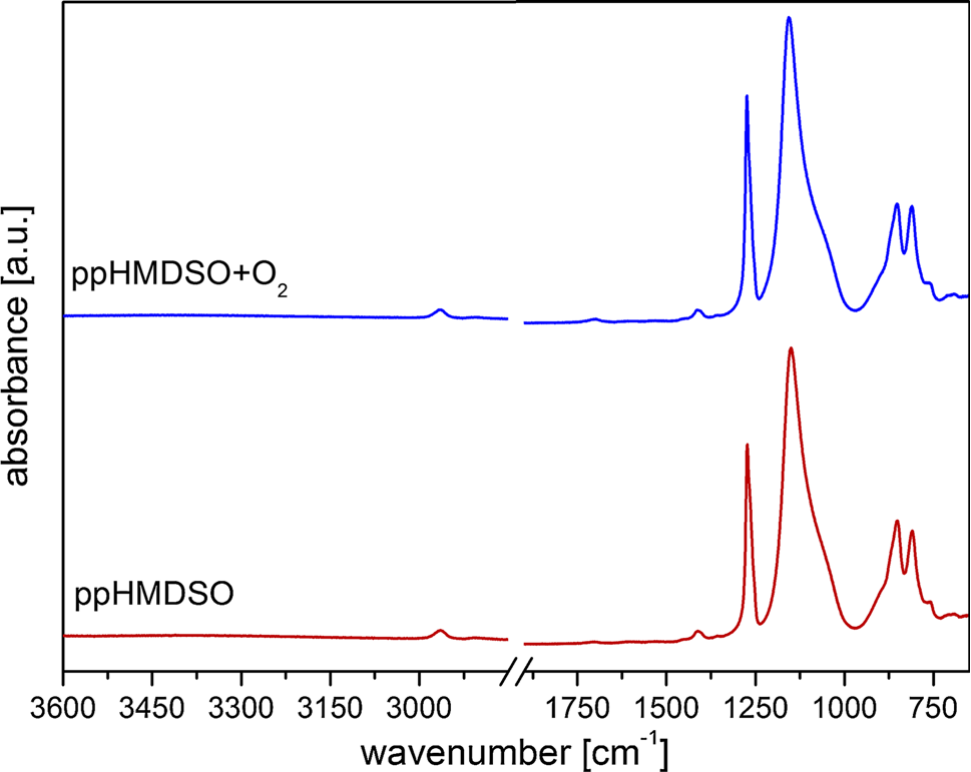
FTIR spectra of ppHMDSO and ppHMDSO + O_2_. FTIR spectra of ppHMDSO (*red line*) and ppHMDS + O_2_ (*blue line*) recorded in the IRRAS mode on gold-coated glass substrates (Color figure online)

Chemical functional groups of the plasma polymerized HMDSO films were analysed by infrared spectroscopy. The IRRAS spectra of ppHMDSO and ppHMDSO + O_2_ deposited films show typical peaks of SiO_x_C_y_H_z_-like plasma polymers deposited from HMDSO in the excess of oxygen. The bands presented in both spectra ppHMDSO and ppHMDSO + O_2_ indicate the presence of Si-(CH_3_)_x_ (where x = 1, 2 and 3), with the stretching vibrations at a wavenumber of 1260 cm^−1^ and Si-(CH_3_)_x_ deformation vibrations at 860 cm^−1^ (Fig. 2). The C–H symmetric and asymmetric stretching vibration of CH in Si-CH_3_ appeared at 2910 and 2966 cm^−1^, CH_3_ bending was observed at 1384 cm^−1^. The main Si–O–Si band appeared at a typical wavenumber of 1180 cm^−1^ with the corresponding stretching vibration at 810 cm^−1^ (Fig. 2).

### X-ray photoelectron spectroscopy (XPS)

The uncoated titanium samples showed the typical elemental surface composition of titanium from the bulk material itself and oxygen, which is mainly due to the oxide layer that is formed on titanium surfaces. The carbon content is due to adsorbed organic molecules, which are always found on oxide surfaces. The small amount silicon originates from fabrication processes of the manufacturer.

XPS analysis of both plasma polymerized HMDSO surfaces showed no signal from the titanium bulk material indicating a complete coverage of the substrate surface after plasma treatment. The films showed only carbon-, oxygen- and silicon signals from the organo-silicon monomer and were free of any surface contamination. Additional oxygen plasma treatment (HMDSO + O_2_) led to an increase in oxygen and decrease in carbon elemental surface composition, while the silicon value remained almost unchanged. Surface elemental compositions (at. %) as well as the resulting elemental ratios are shown in Table 1.

### Cell proliferation

**Fig. 3:**
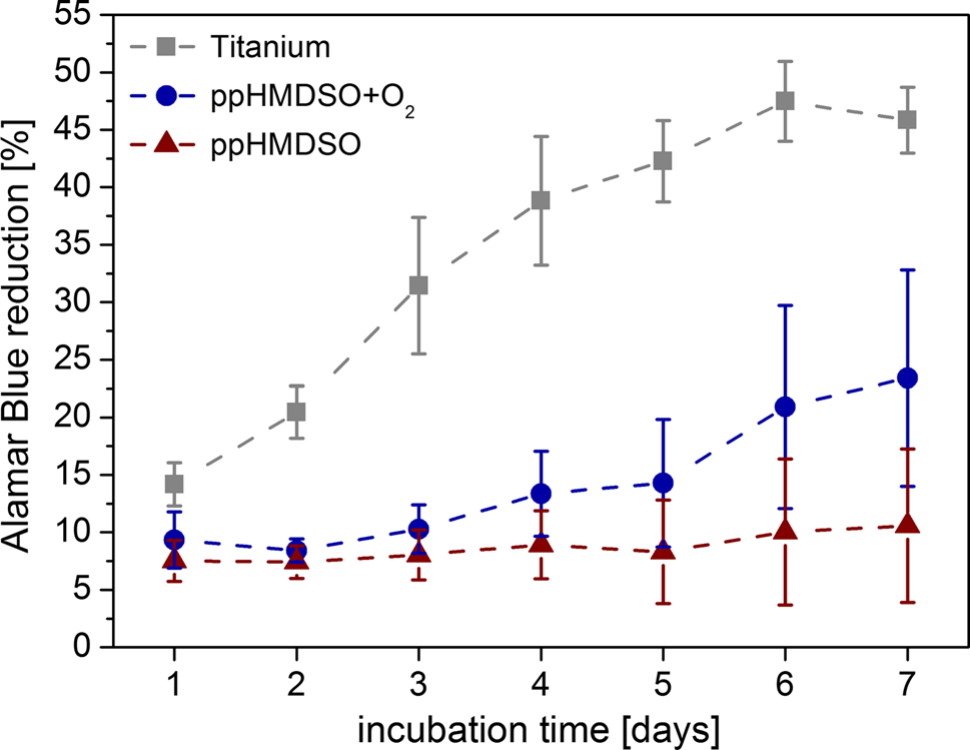
AlamarBlue assay for titanium, ppHMDSO and ppHMDSO + O_2_. Results of the alamarBlue assay of human fibroblasts seeded on titanium (*black*), ppHMDSO (*red*) and ppHMDSO + O_2_ (*blue*) over 7 days. Displayed results show the mean ± the standard deviation (Color figure online)

AlamarBlue assay over 7 days showed different proliferation patterns of fibroblasts on the three analysed surfaces. The fibroblast growth on titanium increased continuously and reached a maximum on day 6. Compared to this titanium reference, the cell growth on both plasma-polymerized HMDSO surfaces was reduced. The effect was most pronounced on the ppHMDSO surface, which showed no relevant proliferation over 7 days (Fig. 3). ppHMDSO + O_2_ showed an increase in proliferation after day 2, however still significantly lower than the titanium reference. Significant differences (Friedman test) existed between the surfaces at all 7 time-points. Paired comparison (Wilcoxon test) yielded *P* values ≤ 0.01 for all surfaces at all points of time. AlamarBlue assay with the progressions of cell proliferation over 7 days are shown in Fig. 3.

### Confocal laser scanning microscopy (CLSM)

**Fig. 4:**
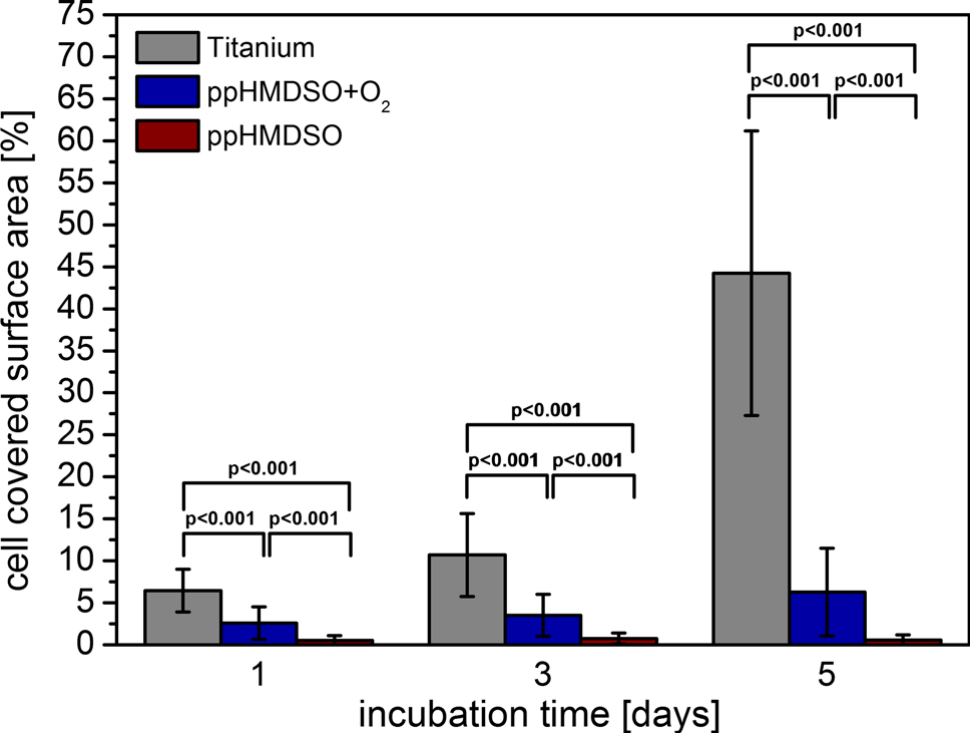
Quantitative analysis of the cell covered surface area for titanium, ppHMDSO and ppHMDSO + O_2_ on day 1, 3 and 5. Displayed results show the mean ± the standard deviation

On titanium the number of cells and cell covered area increased from day 1 over day 3 to day 5. The cells were already elongated and well spread after day 1 with few filopodia at the cell edges. They appeared to preserve this morphology at all times. After 5 days the number of filopodia at the cell periphery had increased. After getting into closer proximity to the neighbouring cells they start forming cell–cell contacts. The fibroblast covered implant surface area increased continuously from day 1 to day 5 (Fig. 4).

Markedly fewer cells were seen on the two plasma modified titanium specimen. On the ppHMDSO surface an unspread morphology of the fibroblasts was observed. After day 1 the fibroblasts were round, most of them with only few filopodia of marginal size. After 3 and 5 days the cell morphology did not differ and only round and unspread or minimally elongated cells were present (Fig. 4). The ppHMDSO + O_2_ modification showed a different morphology. After 1 day few cells were visible on the surface. However besides round unspread cells, elongated fibroblast were detected. Cells displayed filopodia emerging from the cell periphery after 3 and 5 days. While cell number was significantly reduced compared to standard titanium, cell morphology mainly showed an elongated phenotype. The percentage of the total fibroblasts covered area for both plasma-polymerized HMDSO surfaces was markedly reduced compared to the reference titanium. ppHMDSO + O_2_ did show an increase in covered surface area up to 6 % on day 5. Fibroblasts on the ppHMDSO surface did not show an increase in covered surface area with values remaining below 1 % at all time-points (Fig. 4).

Significant differences (Friedman test) existed between the cell covered surface areas at the three different time-points. Paired comparison (Wilcoxon test) yielded *P* values ≤ 0.001 for all surfaces at all time-points.

**Fig. 5:**
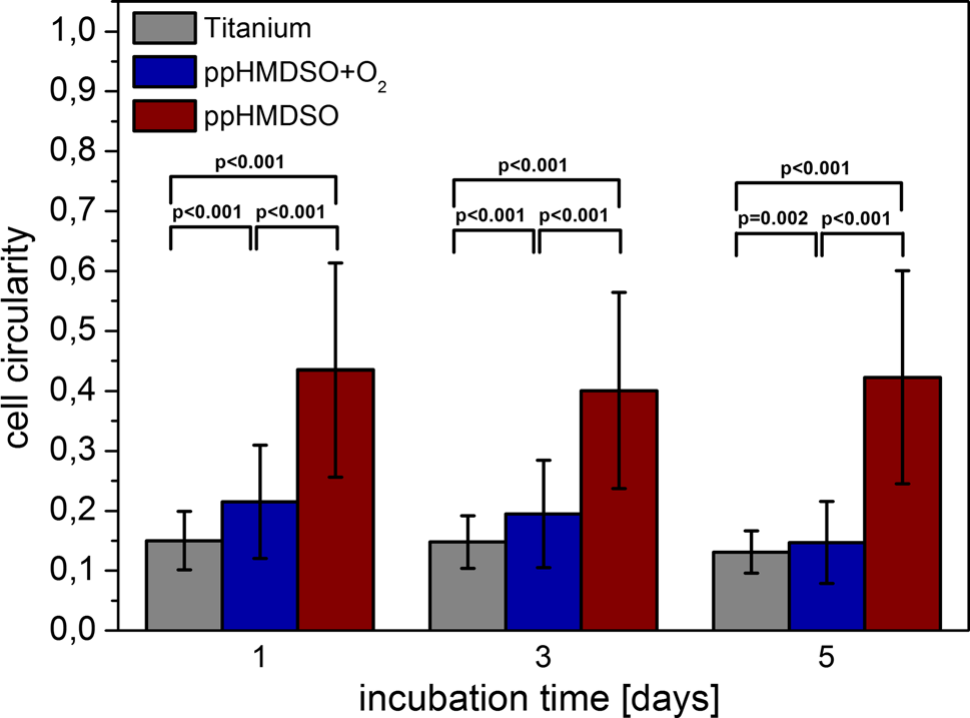
Quantitative analysis of the cell circularity for titanium, ppHMDSO and ppHMDSO + O_2_ on day 1, 3 and 5. Displayed results show the mean ± the standard deviation

The cell circularity as a measure of cell shape showed a clear difference between the three surfaces. On the ppHMDSO surfaces high circularity values of greater 0.4 were observed on day 1, 3, and 5 (Fig. 5). ppHMDSO + O_2_ enhanced cell spreading and elongation and resulted in lower circularity values. Cell circularity values of ppHMDSO + O_2_ were found to be close to unmodified titanium ranging from 0.20 to 0.15. Within each group cell circularity values remained quite constant on day 1, 3, and 5. Quantitative analyses of the cell circularity for titanium, ppHMDSO and ppHMDSO + O_2_ on day 1, 3, and 5 are shown in Fig. 5.

### Surface pattern

**Fig. 6:**
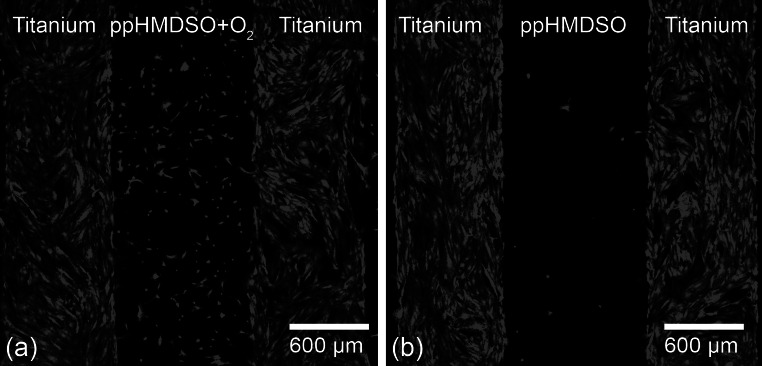
Confocal laser scanning microscopy of eGFP-transducted fibroblasts on ppHMDSO-titanium (**a**) and ppHMDSO + O_2_-titanium (**b**) patterned surface after 5 days

Corresponding to the results shown above for quantitative analyses of cell adhesion it is clearly visible in Fig. 6 that both, fibroblast adhesion and proliferation are mainly limited to the unmodified titanium on patterned surfaces. The clear edges demonstrate the abrupt change from titanium to ppHMDSO (Fig. 6a) and ppHMDSO + O_2_ (Fig. 6b) modified surface areas. However, this effect was strongly pronounced in the surface area modified by ppHMDSO, which only revealed very few rounded cells.

### Imaging of cells from the supernatant

**Fig. 7:**
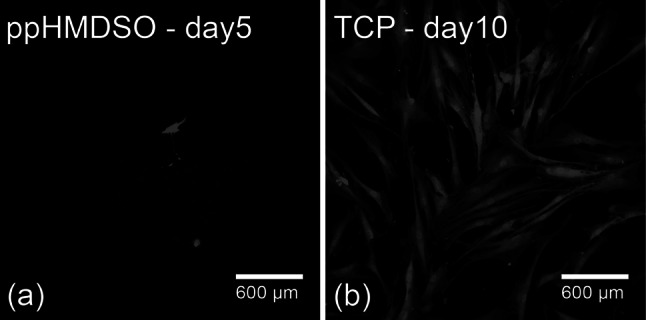
Confocal Laser Scanning Microscopy of the eGFP-transducted NDHF-p adult fibroblasts on ppHMDSO on day 5 (**a**) and after transferring the supernatant to TCP on day 10 (**b**)

After 5 days of incubation on the most anti-adhesive modification ppHMDSO (Fig. 7a), cells from the supernatant transferred onto standard TCP showed the typical spindle-shaped, flatten phenotype of fibroblasts with formation of filopodia and cell–cell contacts on day 10 (Fig. 7b). Additionally the fibroblasts displayed an increasing proliferation.

### Annexine V/propidium iodide assay

**Fig. 8:**
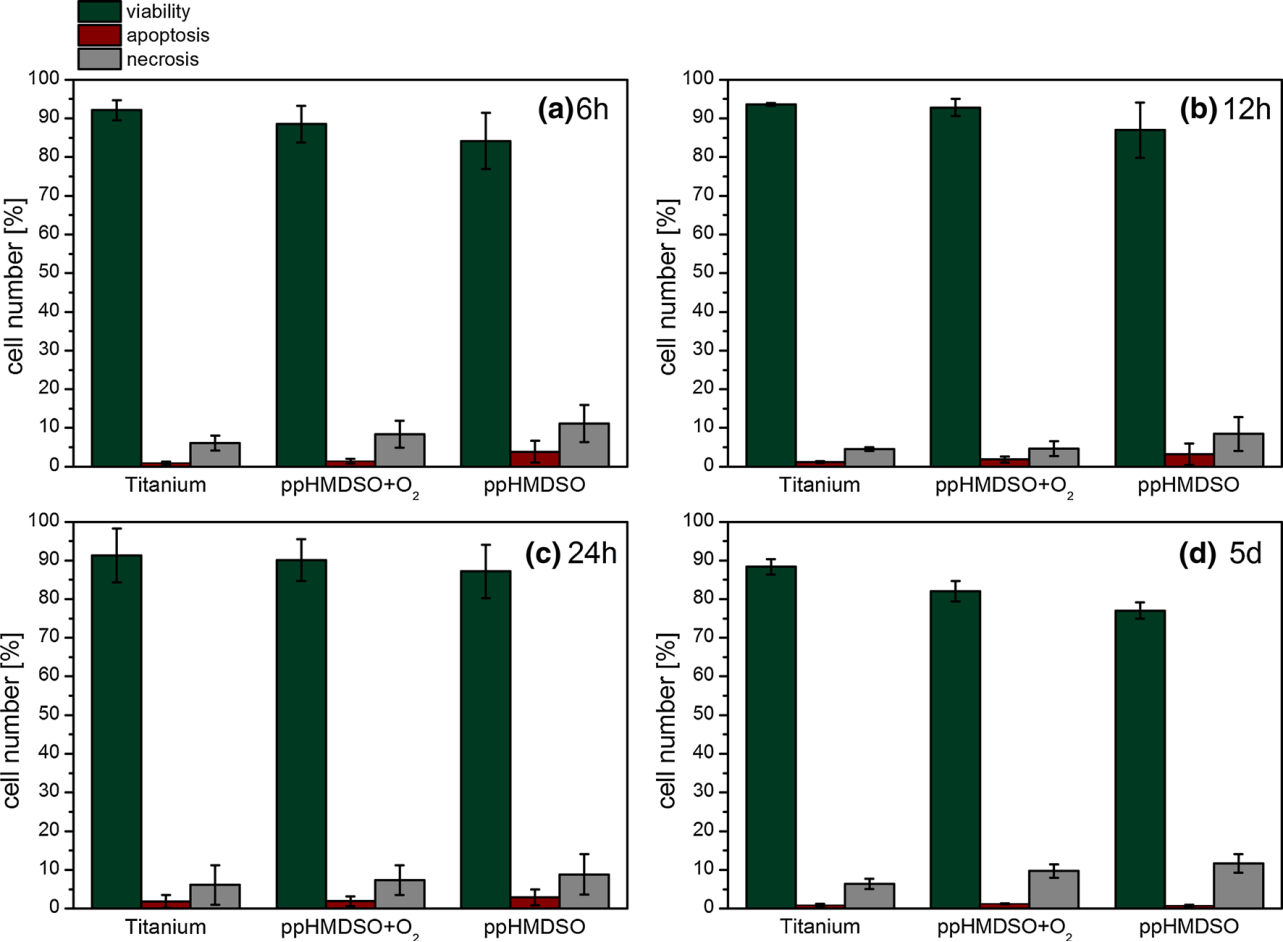
Results of the Annexine V/propidium iodide assay for viability, apoptosis and necrosis at 6h h (**a**), 12 h (**b**), 24 h (**c**) and 5 days (**d**)

Although the cell numbers in ppHMDSO and ppHMDSO + O_2_ groups were significantly lower in comparison to the titanium reference, the viability of the detected cells remained quite constant over the first 24 h on each of the surfaces. The highest viability values were recorded on titanium, followed by ppHMDSO + O_2_ and ppHMDSO. There was also a trend to lower cell viability after 5 days for the HMDSO modified surfaces and on ppHMDSO + O_2_ compared to titanium (Fig. 8).

There was a trend to higher apoptosis for ppHMDSO compared to ppHMDSO + O_2_ and titanium. Within each group apoptosis values remained quite constant over the first 24 h. On day 5 all three surfaces showed low values for apoptosis around 1 %.

There was a trend to higher cell necrosis at all four time-points for ppHMDSO compared to ppHMDSO + O_2_ and titanium.

The detailed results of the Annexine V/propidium iodide assay are shown in Fig. 8.

## Discussion

Surface properties determine the molecular events at the implant to soft tissue interface. In respect to fibroblasts adhesion, reduced surface roughness of titanium implants has currently proven the greatest effect in vitro and in vivo [7–9]. In the current work, profilometric measurements revealed no significant differences between plasma-modified and unmodified surfaces, demonstrating that the large-scale topography of the clinically used titanium surface remained unchanged. SEM, profilometry, IRRAS, XPS and contact angle measurements were in accordance with previously published data and demonstrated a homogeneous surface modification with the two HMDSO plasma variants [10, 12, 20–22]. The IRRAS spectra of the two HMDSO variants were almost identical. The very short oxygen activation in the ppHMDSO + O_2_ variant modified only the uppermost layer of a plasma polymer surface and cannot be resolved by this technique, as IRRAS probes the full thickness of the plasma film. Oxygen incorporation can however be confirmed from the significant decreasing in water contact angle and the increasing oxygen at % by XPS data, as XPS detects the uppermost 10 nm of the surface atomic layer in the applied mode with a take off angle of 90°. These observations were in accordance to previous published data and results obtained from our group using the same equipment [12, 22–24].

The surface elemental composition and relative ratios of the elements for the surfaces were markedly changed by HMDSO plasma polymerization and thus surface chemistry and surface wettability. XPS analyses for both plasma modifications showed no signal from the titanium bulk material, which indicated a complete coverage of the titanium substrate. Carbon-, oxygen- and silicon signals from the organo-silicon monomer showed varying percentages between the two HMDSO modifications.

Surface wettability is also an important characteristic for regulation of cell attachment and proliferation, which has been changed in the two HMDSO modifications. Many studies have shown the effects of surface wettability on cell behaviour, indicating that cells tend to attach more on hydrophilic than on hydrophobic surfaces [25, 26]. The surface wettability is dependent on the oxygen to HMDSO concentration in the plasma and the post-plasma treatment [27, 28]. It is known that the hydrophobic surface of plasma polymerized HMDSO coatings can be altered to be more hydrophilic by means of an additional short O_2_-plasma treatment [29]. This modification introduces hydrophilic groups into the superficial 1 nm but does not measurably change the surface topography. The two HMDSO surface modifications led to an antithetic change in surface wettability. Clinically used titanium with a mean contact angle of 49° was modified to a more hydrophilic ppHMDSO + O_2_ (37°) and hydrophobic HMDSO (96°) surface. These values are in accordance with literature [17, 18, 27, 28].

The plasma polymer film thickness on the titanium discs was in the range of 50 nm. Coatings of this thickness completely cover substrates surface and are thin enough not to develop large interfacial stress. Moreover a very good adhesion to titanium substrates is achieved due to surface activation prior and during the plasma process [30]. It is well established that the biochemical interaction of a surface with cells is limited to the superficial layer with a maximum thickness of 1 nm [31]. Therefore the cells will not interact with the underlying titanium.

For an in vitro characterization of cell-biomaterial interaction, analyses of cell proliferation and cell morphology cover many relevant biological processes. Like most mesenchymal cells, primary fibroblasts are anchorage-dependent. They require cell anchorage and cell spreading for proliferation, which leads to a modulation of cell morphology and allows for cell growth [32]. However, fibroblasts are more resistant to continued suspension than other cell types, and will reversibly arrest their cell cycle, until they can attach to a surface. Nevertheless, they also will undergo apoptosis if kept in suspension for a prolonged time period. Unmodified titanium surfaces produce the typical spindle-shaped, flatten phenotype of fibroblasts with formation of filopodia, increasing proliferation with confluence and cell–cell contacts. Surface modification by plasma polymerization of HMDSO led to a reduction in cell adhesion and proliferation as detected by CLSM visualization and alamarBlue assay. The alamarBlue assay demonstrated a limited fibroblast proliferation on ppHMDSO + O_2_ compared to titanium. On visualization, fibroblasts on ppHMDSO + O_2_ showed a reduced cell number and elongated phenotype. The total cell covered area was significantly lower than on titanium, but still showed an increase over time. On the ppHMDSO surface no increase in cell number was detected after 7 days. Most notable, fibroblasts cultured on ppHMDSO displayed a rounded, non-spread, non-elongated morphology. In CLSM analysis the cell surface area did not increase over the 5 days. The inability to adhere reversibly arrested their cell cycle and led to a significantly reduced initial cell number, proliferation over time and will eventually lead to cell death [32].

CLSM visualization only showed adherent cells on the surface. Annexine V/propidium iodide assay included cells which had been adherent to the surface as well as cells from the supernatant, which contains most cells in both HMDSO-groups. Cells in the supernatant can be viable but unable to proliferate, resulting in high values of viable cells even in the ppHMDSO group. On both HMDSO modified surfaces, but especially on the ppHMDSO variant, there was a higher apoptosis and necrosis rates at all time-points.

The difference in fibroblast adhesion, proliferation and vitality in our experimental setup can be attributed to the change in surface chemistry since the mechanical properties, roughness and topography of the titanium were preserved. Both modified surfaces (ppHMDSO and ppHMDSO + O_2_) were favourable in regard to preventing fibroblast colonization and proliferation on the surface compared to standard titanium. However, this effect was strongly pronounced on the ppHMDSO surface, which showed reduced adhesion, no relevant proliferation, and no increase in cell covered surface area over the observation period. In regard to a possible clinical application of the ppHMDSO surface, the use as a modification for titanium fracture implants seems like a logical option. Currently no clinically proven surface modification exists, which reduces soft tissue adhesion to the fracture implant. In literature, the most investigated experimental modifications are based on polished pure titanium and titanium molybdenum alloy with a highly reduced surface roughness. They have demonstrated reduced cell adhesion in vitro and a reduced tissue adhesion in an in vivo animal model [7–9, 33–35]. Our results suggest that similar effects can be achieved by changing the chemistry of already existing clinically used titanium implant surfaces by a simple plasma modification of the monomer HMDSO.

## Conclusion

Clinically used titanium surfaces can be readily modified by plasma-assisted deposition of HMDSO thin films. This offers the possibility to selectively engineer an anti-adhesive and anti-proliferative surface, while mechanical properties, roughness and topography of the specimen remain mainly unchanged. The differences in fibroblast adhesion, proliferation, and viability in our experimental setup can therefore be attributed to the change in surface chemistry. Both modified surfaces (ppHMDSO and ppHMDSO + O_2_) were favourable in regard to preventing fibroblast colonization and proliferation on the surface compared to standard titanium. However, this effect was strongly pronounced on the hydrophobic, carbon-rich ppHMDSO surface, which showed reduced adhesion, no significant proliferation and no increase in cell covered surface area over the observation period. Therefore plasma polymerized organosilicon coatings from HMDSO are promising candidates for future developments in anti-adhesive and anti-proliferative implant coatings.
